# Supplementary material for: Developing a Blood Cell‐Based Diagnostic Test for Myalgic Encephalomyelitis/Chronic Fatigue Syndrome Using Peripheral Blood Mononuclear Cells
Source: Adv Sci (Weinh). 2023 Aug 31;10(30):2302146. doi: 10.1002/advs.202302146 (PMC10602530; doi:10.1002/advs.202302146)
Supplement: Supplementary file 1 — Supporting Information [file ADVS-10-2302146-s001.pdf]

ADVANCED SCIENCE
Open Access

Supporting Information

for *Adv. Sci.*, DOI 10.1002/advs.202302146

Developing a Blood Cell-Based Diagnostic Test for Myalgic Encephalomyelitis/Chronic Fatigue Syndrome Using Peripheral Blood Mononuclear Cells

Jiabao Xu, Tiffany Lodge, Caroline Kingdon, James W. L. Strong, John Maclennan, Eliana Lacerda, Slawomir Kujawski, Pawel Zalewski, Wei E. Huang and Karl J. Morten**

Supporting Information for

Developing a blood cell-based diagnostic test for myalgic encephalomyelitis/chronic fatigue syndrome using peripheral blood mononuclear cells

Jiabao Xu, Tiffany Lodge, Caroline Kingdon, James W L Strong, John Maclennan, Eliana Lacerda,Slawomir Kujawski, Pawel Zalewski, Wei E. Huang* and Karl J. Morten*

Correspondence to: Wei E. Huang and Karl J. Morten
Email: karl.morten@wrh.ox.ac.uk and wei.huang@eng.ox.ac.uk

This PDF file includes:

Figures S1 to S2
Tables S1 to S5

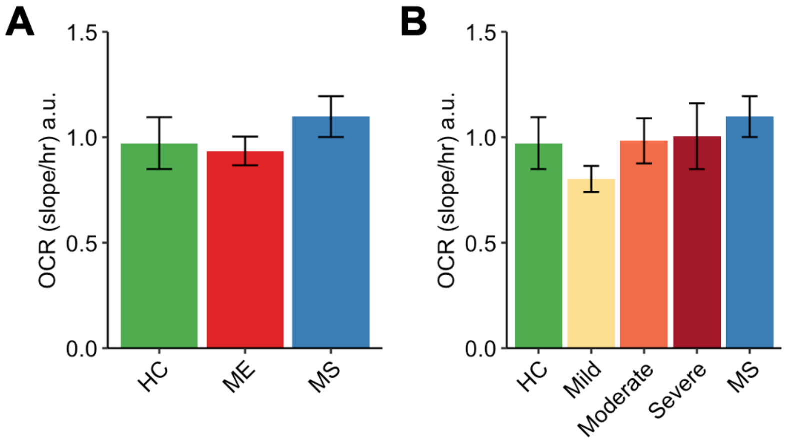

Fig. S1. OCR of PBMCs from 41 human subjects of **(A)** HCs (n = 9), ME patients (n = 26) and MS (n = 6) patients, and **(B)** HCs and MS patients, with ME patients separated based on disease severities of Mild (n = 9), Moderate (n = 8), and Severe (n = 9). No significant statistical difference between HCs and patients were observed.

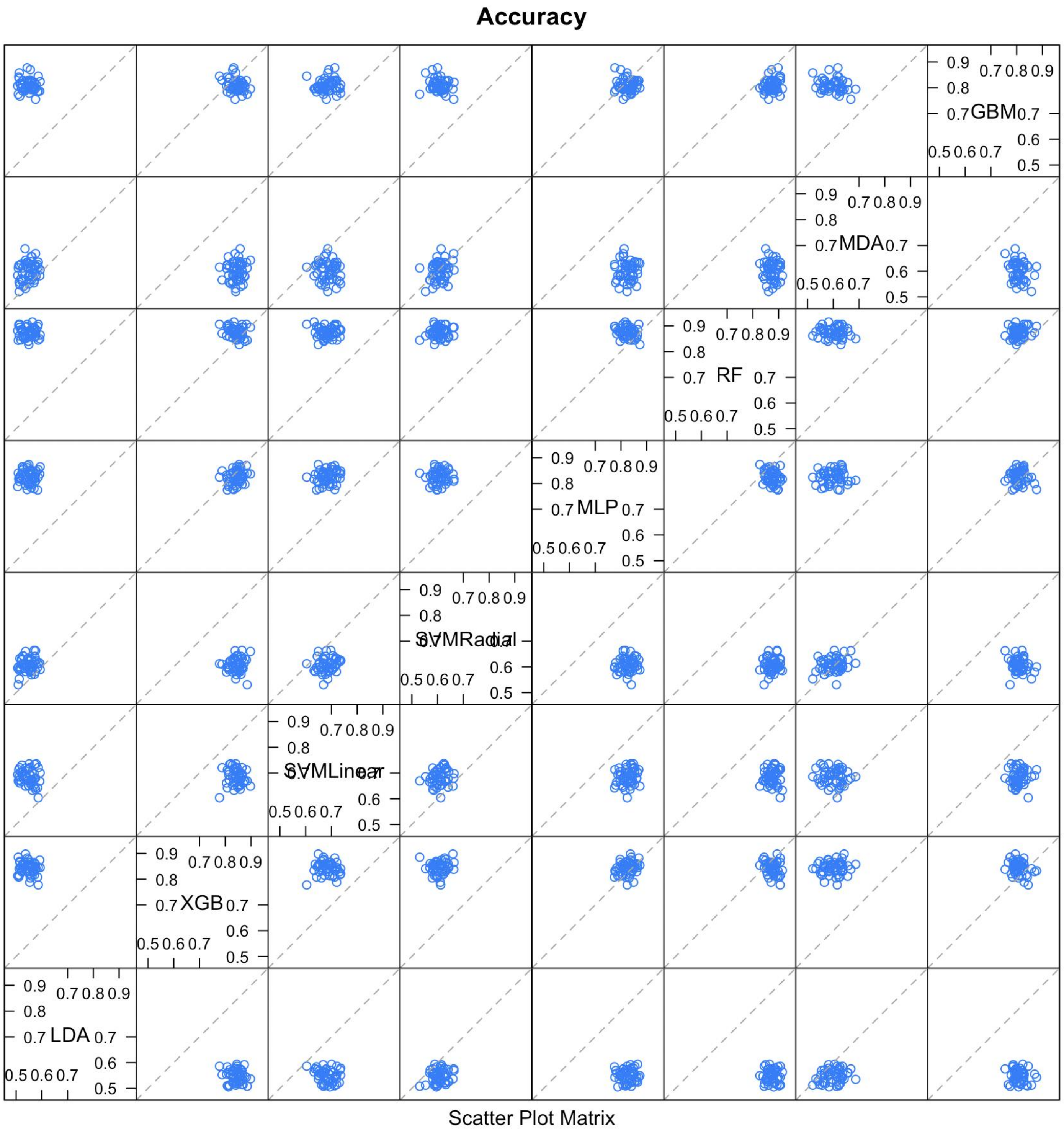

Fig. S2. Model correlations by comparing model accuracies. LDA: Linear Discriminant Analysis; kNN: k-Nearest Neighbour; SVM-Linear: Support Vector Machine with Linear Kernel: SVM-Radial: Support Vector Machine with Radial Basis Function Kernel; MLPNN: Monotone Multi-Layer Perceptron Neural Network; RF: Random Forest; MDA: Mixture Discriminant Analysis; XGB: Extreme Gradient Boosting; GBM: Stochastic Gradient Boosting

Table S1. Retrospective subject cohort breakdown with fatigue groups in this study. Gender: F represents Female and M represents Male; Fatigue Severity Scale (FSS), General Health Questionnaire 28 (GHQ-28). Statistical comparison was shown in the blue area. Comparisons of 2 groups was performed using the Mann Whitney U test (Wilcoxon rank sum test with continuity correction). Comparisons of greater than 2 groups employed the non-parametric Kruskal Wallis rank sum test. Fischer's exact test was used to compare categorical variables. Comparisons with statistical significance where $p < 0.05$ were highlighted.

Variable	*Gender (M/F)*	*Age*	*Disease Duration (years)*	*Body Mass Index*	*FSS*	*Somatic Symptoms(GHQ-28:1-7)*	*Anxiety & Insomnia(GHQ-28:8-14)*	*Social Dysfunction(GHQ-28:15-21)*	*Severe depression(GHQ-28:22-28)*	*GHQ-28:1-28 (Sum)*
ME (Mild) Median	4/20	46	12.4	25.5	57	2.5	1.5	1	0	4
Range	0-1	18-59	0.42-37.9	17.9-46.7	44-63	0-7	0-6	0-6	0-7	0-26
ME (Moderate) Median	1/11	45.5	9	32.4	62.5	3	2	4	0	8
Range	0-1	30-60	1.58-29.2	25.1-53.8	44-63	0-5	0-5	0-7	0-6	0-17
ME (Severe) Median	6/14	40	15.1	21.5	59.5	2.5	1	1.5	0	6.5
Range	0-1	23-59	1.5-39.8	17.2-32.2	54-63	0-7	0-6	0-7	0-5	0-23
ME (All/Pooled) Median	11/45	42.5	12.4	26.2	59	3	1	2	0	7
Range	0-1	18-60	0.42-39.8	17.2-53.9	44-63	0-7	0-6	0-7	0-7	0-26
Multiple Sclerosis Median	6/16	51.5	15.5	23.8	54	3	1	3	0	9
Range	0-1	38-60	1.17-39.2	20.7-37.7	16-63	0-7	0-7	0-7	0-6	0-25
Healthy Controls Median	6/12	43	/	26.6	17	0	0	0	0	0
Range	0-1	18-60	/	20.2-36.4	11-37	0-3	0-3	0-4	0-1	0-8
Across ME Severities (p value)	0.36	0.7601	0.5446	**0.0028**	**0.0667**	0.5764	0.6049	0.2482	0.9371	0.7765
ME (All severities) vs MS (p value)	0.55	**0.0098**	0.2898	0.3141	**0.0017**	0.53	0.8275	0.2532	0.6404	0.3546
Healthy Controls vs ME (All severities) p value	0.3331	1		0.9949	**0**	0.0022	9e-04	0.0119	**0.0287**	5e-04
Healthy Controls vs Multiple Sclerosis (MS) p value	0.7385	**0.0238**		0.2793	**0**	9e-04	0.0036	0.0014	**0.0225**	0

Table S2. UKMEB symptom burden assessment. Symptom feature inclusion was determined by calculating the relative mean ordinal intensity for each variable to provide sufficient detail to rank order (as opposed to a median derived integer with categorical representation) symptoms, allowing for selective inclusion with a >1.5-fold difference between groups (severe ME compared to MS). Air hunger: adjusted for confounding administration of Asthma medications salbutamol and or a steroidal inhaler (n=7 subjects across experimental groups): 0.000251253 (p), 0.008040096 (p) (Hochberg adjusted for multiple comparisons).

UKMEB Full Variable	UKMEB Short Name	Relative ordinal score (Severe ME compared to MS)	Mean ordinal score (HCs)	Range ordinal score (HCs)	NA Count total (%) (HCs)	Mean ordinal score (MS)	Range ordinal score (MS)	NA Count total (%) (MS)	Mean ordinal score (Severe ME)	Range ordinal score (Severe ME)	NA Count total (%) (Severe ME)	p-value (Fisher's Exact Test)	p-value (Hochberg adjusted)
PQ4.9.42 Over past week, have you had worsening symptoms after exertion?	Symptoms worsening after exertion	2.16	0.06	0 - 1	1 (5.56)	1.32	0 - 3	0 (0)	2.85	2 - 3	0 (0)	**0.000001**	**0.000019**
PQ4.9.8 Over past week, have you felt ill (>24hrs) after exertion?	Feeling ill (>24hrs) after exertion	2.59	0.00	0 - 0	1 (5.56)	0.77	0 - 2	0 (0)	2.00	2 - 2	0 (0)	**0.000004**	**0.000112**
PQ4.9.41 Over past week, have you had exercise intolerance?	Exercise intolerance	1.77	0.06	0 - 1	1 (5.56)	1.64	0 - 3	0 (0)	2.90	2 - 3	0 (0)	**0.000013**	**0.000400**
PQ4.9.9 Over past week, have you had pain (>24hrs) after exertion?	Pain (>24hrs) after exertion	2.89	0.06	0 - 1	1 (5.56)	0.76	0 - 2	1 (4.55)	2.20	0 - 3	0 (0)	**0.000023**	**0.000653**
PQ4.9.6 Over past week, have you had new sensitivities?	New sensitivities	4.03	0.06	0 - 1	1 (5.56)	0.55	0 - 3	0 (0)	2.20	0 - 3	0 (0)	**0.000042**	**0.001181**
PQ4.9.19 Over past week, have you had air hunger?	Air hunger	3.80	0.00	0 - 0	1 (5.56)	0.50	0 - 3	0 (0)	1.90	0 - 3	0 (0)	**0.000079**	**0.002133**
PQ4.9.4 Over past week, have you had tender glands in neck/arm pit?	Tender glands in neck/arm pit	4.95	0.06	0 - 1	2 (11.11)	0.33	0 - 3	1 (4.55)	1.65	0 - 3	0 (0)	**0.000106**	**0.002753**
PQ4.9.39 Over past week, have you had	Fatigue (>24hrs) after exertion	1.52	0.00	0 - 0	1 (5.56)	1.32	0 - 2	0 (0)	2.00	2 - 2	0 (0)	**0.000204**	**0.005101**

fatigue (>24hr) after exertion?												
PQ4.9.21 Over past week, have you had new headaches?	New headaches	2.05	0.00	0 - 0	1 (5.56)	1.00	0 - 3	0 (0)	2.05	0 - 3	1 (5)	**0.000261**	**0.006258**
PQ4.7b In past week, how likely are you to doze off while watching tv?	Likelihood of dozing off while watching TV	0.34	1.24	0 - 3	1 (5.56)	2.18	0 - 3	0 (0)	0.75	0 - 3	0 (0)	**0.000494**	**0.011367**
PQ4.9.35 Over past week, have you had slow thinking?	Slow thinking	1.56	0.18	0 - 1	1 (5.56)	1.45	0 - 3	0 (0)	2.26	2 - 3	1 (5)	**0.000711**	**0.015641**
PQ4.9.2 Over past week, have you had flu symptoms?	Had flu symptoms	4.63	0.29	0 - 2	1 (5.56)	0.27	0 - 3	0 (0)	1.26	0 - 3	1 (5)	**0.002296**	**0.048215**
PQ4.9.23 Over past week, have you had sensitivity to light/noise/smell/touch?	Sensitivity to noise/smell/touch	2.31	0.12	0 - 2	1 (5.56)	0.91	0 - 3	0 (0)	2.10	0 - 3	0 (0)	**0.002787**	0.055738
PQ4.9.37 Over past week, have you had unrefreshing sleep?	Unrefreshing sleep	1.67	0.41	0 - 1	1 (5.56)	1.50	0 - 3	0 (0)	2.50	1 - 3	0 (0)	**0.011295**	0.205433
PQ4.9.10 Over past week, have you had muscle pain?	Muscle pain	1.56	0.47	0 - 2	1 (5.56)	1.38	0 - 3	1 (4.55)	2.16	0 - 3	1 (5)	**0.011413**	0.205433
PQ4.9.28 Over past week, have you had short term memory problems?	Short term memory problems	1.55	0.29	0 - 2	1 (5.56)	1.45	0 - 3	0 (0)	2.25	1 - 3	0 (0)	**0.013710**	0.233064
PQ4.9.1 Over past week, have you had a sore throat?	Sore throat	2.57	0.35	0 - 3	1 (5.56)	0.41	0 - 3	0 (0)	1.05	0 - 3	1 (5)	**0.017284**	0.233719
PQ4.7c In past week, how likely are you to doze off while sitting inactive?	Likelihood of dozing off while sitting inactive	0.31	0.24	0 - 1	1 (5.56)	1.59	0 - 3	0 (0)	0.50	0 - 3	0 (0)	**0.018914**	0.233719

PQ4.9.47 Over past week, have you had palpitations other times?	Palpitations at times other than while standing	2.97	0.09	0 - 1	7 (38.89)	0.45	0 - 2	0 (0)	1.35	0 - 3	0 (0)	**0.025242**	0.233719
PQ4.9.50 Over past week, have you been unusually sweaty?	Been unusually sweaty	3.54	0.24	0 - 1	1 (5.56)	0.41	0 - 3	0 (0)	1.45	0 - 3	0 (0)	**0.025886**	0.233719
PQ4.9.7 Over past week, have you had alcohol intolerance?	Alcohol intolerance	2.56	0.08	0 - 1	6 (33.33)	0.64	0 - 3	0 (0)	1.63	0 - 3	1 (5)	**0.041215**	0.233719
PQ4.9.46 Over past week, have you had palpitations while standing?	Palpitations while standing	3.46	0.06	0 - 1	1 (5.56)	0.32	0 - 2	0 (0)	1.10	0 - 3	0 (0)	**0.043068**	0.233719
PQ4.9.15 Over past week, have you had pain in >=2 joints?	Pain in 2 or more joints	1.65	0.29	0 - 2	1 (5.56)	1.09	0 - 3	0 (0)	1.80	0 - 3	0 (0)	**0.044137**	0.233719
PQ4.9.31 Over past week, have you had disorientation?	Disorientation	2.04	0.18	0 - 2	1 (5.56)	0.77	0 - 2	0 (0)	1.58	0 - 3	1 (5)	0.053621	0.233719
PQ4.9.14 Over past week, have you had pain in chest/abdomen?	Pain in chest/abdomen	1.95	0.08	0 - 1	6 (33.33)	0.73	0 - 3	0 (0)	1.42	0 - 3	1 (5)	0.080233	0.233719
PQ4.9.45 Over past week, have you had dizziness while standing?	Dizziness while standing	1.75	0.12	0 - 1	1 (5.56)	1.00	0 - 3	0 (0)	1.75	0 - 3	0 (0)	0.084262	0.233719
PQ4.9.48 Over past week, have you felt light-headed?	Felt light-headed	1.98	0.18	0 - 1	1 (5.56)	0.86	0 - 3	1 (4.55)	1.70	0 - 3	0 (0)	0.091235	0.233719
PQ4.9.49 Over past week, have you been extremely pale?	Been extremely pale	2.20	0.12	0 - 2	1 (5.56)	0.73	0 - 3	0 (0)	1.60	0 - 3	0 (0)	0.096796	0.233719
PQ4.9.3 Over past week, have you had fever/chills?	Had fever/chills	2.89	0.18	0 - 2	1 (5.56)	0.45	0 - 3	0 (0)	1.32	0 - 3	1 (5)	0.097901	0.233719
PQ4.9.5 Over past week, have you had any viral infections?	Any viral infections	3.24	0.13	0 - 2	2 (11.11)	0.23	0 - 2	0 (0)	0.74	0 - 3	1 (5)	0.131622	0.233719

PQ4.9.57 Over past week, have you had unintentional weight changes?	Unintentional weight changes	1.93	0.36	0 - 2	7 (38.89)	0.91	0 - 3	0 (0)	1.75	0 - 3	0 (0)	0.182924	0.233719
PQ4.9.18 Over past week, have you had back weakness?	Back weakness	1.56	0.29	0 - 1	1 (5.56)	1.09	0 - 3	0 (0)	1.70	0 - 3	0 (0)	0.233719	0.233719

Table S3. Pearson correlation coefficient matrix measuring correlations between pairs of variables from the Raman LDA model and potential confounders; correlations with values > 0.5 were highlighted.

		A	B	C	D	E	F	G	H	I	J	K	L	M	N	O	P	Q	R
**LD1**	**A**	**1**	-0.48	-0.02	0.04	0.18	-0.18	0.17	-0.21	-0.05	-0.24	-0.15	0.3	-0.18	-0.13	-0.02	-0.08	-0.06	0.01
**LD2**	**B**	-0.48	**1**	-0.06	-0.14	-0.2	0.06	-0.22	0.06	-0.03	-0.06	0.19	-0.27	0.29	0.38	0.11	0.09	0.15	0
**Medicine last 3 months (Y/N)**	**C**	-0.02	-0.06	**1**	**0.56**	0.16	0.34	0.19	0.07	0.19	0.09	0.14	0.1	-0.09	-0.08	-0.06	0.23	0.09	0.04
**Medicine current (Y/N)**	**D**	0.04	-0.14	**0.56**	**1**	0.2	0.41	0.3	0.14	0.2	0.15	0.17	0.19	-0.19	-0.13	-0.24	0.11	0.06	-0.05
**Supplement Count**	**E**	0.18	-0.2	0.16	0.2	**1**	-0.14	**0.64**	-0.15	-0.26	-0.2	-0.11	0.09	0.02	0.07	-0.34	0.09	0.02	-0.21
**Medication Count**	**F**	-0.18	0.06	0.34	0.41	-0.14	**1**	-0.19	0.41	0.32	0.49	0.39	0.36	-0.13	-0.02	0.14	0	0.04	0.07
**Supplements Present**	**G**	0.17	-0.22	0.19	0.3	**0.64**	-0.19	**1**	-0.18	-0.18	-0.14	-0.23	-0.01	0.03	-0.21	-0.44	-0.14	0.09	-0.18
**Medication_Class_Gabapentinoid_Present**	**H**	-0.21	0.06	0.07	0.14	-0.15	0.41	-0.18	**1**	0.27	0.42	0.24	-0.08	-0.16	0.1	0	-0.05	-0.11	-0.07
**Medication_Class_NSAID_Paracetamol_Present**	**I**	-0.05	-0.03	0.19	0.2	-0.26	0.32	-0.18	0.27	**1**	**0.59**	-0.01	0.02	0.05	-0.11	0.2	0.16	0.02	0.12
**Medication_Class_Opiate_Present**	**J**	-0.24	-0.06	0.09	0.15	-0.2	0.49	-0.14	0.42	**0.59**	**1**	0.12	-0.01	0	-0.13	0.02	0.07	-0.19	-0.01
**Medication_Class_SSRI_SNRI_Present**	**K**	-0.15	0.19	0.14	0.17	-0.11	0.39	-0.23	0.24	-0.01	0.12	**1**	-0.08	-0.23	0.13	0.15	-0.11	-0.1	0.07
**Medication Class Tricyclic Or Mirtazapine Present**	**L**	0.3	-0.27	0.1	0.19	0.09	0.36	-0.01	-0.08	0.02	-0.01	-0.08	**1**	-0.18	0.11	0	0.13	-0.01	0.17
**Sex**	**M**	-0.18	0.29	-0.09	-0.19	0.02	-0.13	0.03	-0.16	0.05	0	-0.23	-0.18	1	0.02	-0.07	-0.01	0.25	-0.13
**Age at survey**	**N**	-0.13	0.38	-0.08	-0.13	0.07	-0.02	-0.21	0.1	-0.11	-0.13	0.13	0.11	0.02	**1**	0.04	0.37	0.06	0.14
**BMI**	**O**	-0.02	0.11	-0.06	-0.24	-0.34	0.14	-0.44	0	0.2	0.02	0.15	0	-0.07	0.04	**1**	-0.03	0.04	0.34
**Disease duration (years)**	**P**	-0.08	0.09	0.23	0.11	0.09	0	-0.14	-0.05	0.16	0.07	-0.11	0.13	-0.01	0.37	-0.03	**1**	0.1	-0.07
**Processing duration (seconds)**	**Q**	-0.06	0.15	0.09	0.06	0.02	0.04	0.09	-0.11	0.02	-0.19	-0.1	-0.01	0.25	0.06	0.04	0.1	**1**	0.09
**Recruiting time/freezing duration (days)**	**R**	0.01	0	0.04	-0.05	-0.21	0.07	-0.18	-0.07	0.12	-0.01	0.07	0.17	-0.13	0.14	0.34	-0.07	0.09	**1**

Table S4. Top Raman peak features selected based on descending LDA contribution. At each Raman peak, averaged quantification of HC, ME and MS is shown. Statistical comparison is shown as p value by a global student t-test.

Raman Peak (cm^{-1})	***LDA contribution***	***HC average***	***ME average***	***MS average***	***P value***
**467.324**	285.43	0.212	0.201	0.109	3.42E-10
**1180.3**	279.03	1.501	1.458	1.506	0.03603478
**1366.86**	276.80	2.203	2.242	2.34	1.02E-05
**1173.68**	255.38	1.622	1.59	1.663	0.0226378
**742.745**	255.23	1.374	1.35	1.462	0.0016585
**3054.15**	253.30	1.258	1.261	1.202	0.00018715
**2810.91**	237.08	0.256	0.28	0.278	0.11974009
**520.949**	231.16	0.487	0.495	0.41	2.85E-05
**1631.46**	220.60	1.13	1.128	1.095	0.45513773
**431.398**	218.41	0.448	0.432	0.3	8.01E-10
**1347.41**	191.05	2.37	2.434	2.505	9.06E-07
**460.15**	186.97	0.254	0.223	0.137	9.73E-10
**1080.47**	186.61	1.922	1.907	1.953	0.12243916
**1700.24**	179.91	0.656	0.632	0.525	0.00016772
**1482.64**	179.09	1.525	1.565	1.642	0.00402072
**1587.41**	175.63	1.196	1.235	1.247	0.32159906
**3030.43**	171.42	1.167	1.149	1.109	0.00370214
**556.523**	165.11	0.509	0.539	0.448	0.00016625
**3062.04**	155.37	1.189	1.195	1.135	0.00012515
**348.223**	155.31	0.823	0.785	0.701	1.55E-05
**1130.53**	154.44	1.583	1.56	1.642	0.13577574
**395.327**	152.81	0.603	0.587	0.443	1.19E-06
**416.986**	150.81	0.639	0.622	0.481	3.13E-08
**2824.48**	146.38	0.474	0.496	0.518	0.05868149
**373.617**	142.63	0.579	0.548	0.427	1.30E-07
**1023.39**	142.23	1.588	1.543	1.564	0.0402755
**794.753**	140.72	1.142	1.128	1.09	0.05205117
**1389.5**	137.58	1.519	1.551	1.596	0.03224369
**2859.63**	132.97	3.523	3.545	3.742	0.00236603
**1536.77**	128.30	0.572	0.582	0.588	0.91035318
**770.519**	119.92	1.382	1.357	1.323	0.11458769
**767.054**	118.03	1.157	1.138	1.093	0.00822416
**1489.03**	116.36	1.155	1.172	1.22	0.20345991
**577.799**	115.99	0.374	0.375	0.293	0.0001191
**2851.53**	114.21	2.692	2.7	2.88	0.00628556
**1408.85**	112.36	1.454	1.477	1.492	0.20159483

**2988.08**	109.69	4.008	4.047	4.061	0.5193555
**2800.04**	109.02	0.19	0.202	0.2	0.66904405
**427.797**	108.79	0.497	0.478	0.342	3.48E-10
**972.718**	108.42	1.602	1.641	1.575	0.12771077
**3056.78**	107.30	1.262	1.281	1.222	0.00011284
**495.963**	107.08	0.474	0.471	0.411	0.00162004
**1428.16**	106.76	1.874	1.901	1.978	7.74E-05
**1026.76**	103.74	1.741	1.705	1.704	0.1179658
**1524.07**	103.61	0.777	0.739	0.806	0.34013404
**753.17**	103.19	1.102	1.103	1.095	0.90952865
**1305.12**	100.76	3.103	3.121	3.28	2.20E-10
**894.445**	98.81	1.446	1.415	1.333	2.35E-06
**1593.72**	97.72	1.04	1.058	1.031	0.62109426
**1590.56**	96.37	1.081	1.111	1.089	0.50027145
**818.92**	96.03	1.213	1.18	1.116	6.12E-07
**641.33**	95.49	0.595	0.614	0.554	0.00220252
**1040.22**	95.30	1.677	1.626	1.521	3.12E-05
**1450.64**	92.45	2.876	2.911	3.041	3.00E-11
**1331.17**	91.13	3.259	3.295	3.443	1.35E-06
**2854.23**	88.61	2.912	2.931	3.124	0.0015022
**860.195**	88.55	1.409	1.357	1.287	7.35E-09
**965.94**	87.58	1.642	1.666	1.598	0.16692987
**1672.17**	86.37	1.748	1.795	1.823	0.02620277
**798.208**	84.25	1.09	1.054	1.002	1.37E-05
**1160.43**	84.21	1.538	1.471	1.542	0.05395872
**2958.83**	84.11	7.167	7.298	7.484	0.01510187
**3022.51**	83.74	1.323	1.32	1.288	0.08256413
**1759.18**	82.15	0.23	0.078	-0.131	5.44E-12
**1469.85**	81.14	1.886	1.914	1.977	0.0026206
**1301.86**	80.60	3.081	3.086	3.241	1.22E-10
**711.395**	80.05	0.702	0.691	0.634	0.00123915
**1090.51**	79.61	2.097	2.09	2.15	0.03577563
**1584.25**	79.09	1.396	1.424	1.47	0.23850419
**1555.8**	75.19	0.866	0.886	0.88	0.70012075
**669.421**	75.05	0.675	0.664	0.617	0.00490924
**2993.39**	75.01	3.313	3.347	3.323	0.49207105
**488.812**	74.82	0.522	0.505	0.447	0.00089672
**506.679**	74.12	0.356	0.35	0.253	1.74E-08
**2865.03**	73.20	4.366	4.408	4.662	0.00030988
**1476.25**	71.44	1.729	1.761	1.832	0.00564951

Table S5. Model performance in accuracy by individual classifiers from the ensemble learner. LDA: Linear Discriminant Analysis; XGB: Extreme Gradient Boosting; SVM-Linear: Support Vector Machine with Linear Kernel: SVM-Radial: Support Vector Machine with Radial Basis Function Kernel; MLPNN: Monotone Multi-Layer Perceptron Neural Network; RF: Random Forest; MDA: Mixture Discriminant Analysis; GBM: Stochastic Gradient Boosting.

Classifier	*Hyperparameters*	*Performance on train set*	*Performance on test set*
**LDA**	**dimension = 4**	**54.8%**	**47.1%**
**XGB**	**nrounds = 150, max_depth = 3, eta = 0.4, gamma = 0, subsample = 1, colsample_bytree = 0.8, rate_drop = 0.01, skip_drop = 0.95 and min_child_weight = 1**	**84.3%**	**51.2%**
**SVM-Linear**	**Cost = 1**	**68.4%**	**48.8%**
**SVM-Radial**	**mtry = 100, splitrule = extratrees, min.node.size = 1**	**60.1%**	**49.9%**
**MLPNN**	**hidden1 = 15, n.ensemble = 5**	**82.6%**	**50.2%**
**RF**	**mtry = 109, splitrule = extratrees, min.node.size = 1**	**87.4%**	**61.2%**
**MDA**	**subclasses = 4**	**60.1%**	**53.1%**
**GBM**	**n.trees = 150, interaction.depth = 3, shrinkage = 0.1, n.minobsinnode = 10.**	**80.9%**	**48.1%**
